# Antidepressants and inflammatory bowel disease: a systematic review

**DOI:** 10.1186/1745-0179-2-24

**Published:** 2006-09-20

**Authors:** Antonina A Mikocka-Walus, Deborah A Turnbull, Nicole T Moulding, Ian G Wilson, Jane M Andrews, Gerald J Holtmann

**Affiliations:** 1 School of Psychology and Discipline of General Practice, University of Adelaide, and Department of Gastroenterology, Hepatology and General Medicine, Royal Adelaide Hospital, level 3, Eleanor Harrald Building, 5005 Adelaide, South Australia, Australia; 2 School of Psychology, the University of Adelaide, level 4, Hughes Building, Adelaide 5005, South Australia, Australia; 3 Nicole T. Moulding, Discipline of General Practice, the University of Adelaide, Level 3, Eleanor Harrald Building, Adelaide 5005, South Australia, Australia; 4 School of Medicine, University of Western Sydney, Locked Bag 1797, Penrith SouthDC 1797, New South Wales, Australia; 5 Department of Gastroenterology, Hepatology and General Medicine, the Royal Adelaide Hospital, North Wing Q7, Adelaide 5005, South Australia, Australia; 6 Department of Gastroenterology, Hepatology and General Medicine, the Royal Adelaide Hospital, North Wing Q7, Adelaide 5005, South Australia, Australia

## Abstract

**Background:**

A number of studies have suggested a link between the patient's psyche and the course of inflammatory bowel disease (IBD). Although pharmacotherapy with antidepressants has not been widely explored, some investigators have proposed that treating psychological co-morbidities with antidepressants may help to control disease activity. To date a systematic analysis of the available studies assessing the efficacy of antidepressants for the control of somatic symptoms in IBD patients has not been performed.

**Methods:**

We searched electronic databases, without any language restriction. All relevant papers issued after 1990 were examined.

**Results:**

12 relevant publications were identified. All of them referred to non-randomised studies. Antidepressants reported in these publications included paroxetine, bupropion, amitriptyline, phenelzine, and mirtazapine. In 10 articles, paroxetine, bupropion, and phenelzine were suggested to be effective for treating both psychological and somatic symptoms in patients suffering from IBD. Amitriptyline was found ineffective for treating somatic symptoms of IBD. Mirtazapine was not recommended for IBD patients.

**Conclusion:**

Although most of reviewed papers suggest a beneficial effect of treatment with antidepressants in patients with IBD, due to the lack of reliable data, it is impossible to judge the efficacy of antidepressants in IBD. Properly designed trials are justified and needed based upon the available uncontrolled data.

## Background

Inflammatory bowel disease (IBD) is a group of relapsing incurable diseases of the gastrointestinal tract. There is ongoing active research to find a curative treatment and understand the aetiology of IBD in immunology [1], microbiology [2], molecular biology [3] and food science [4]. As yet, IBD is incompletely understood (or treated) and the individual patient's response to treatment is variable. Until globally effective treatment is found, there is a need to decrease the number of relapses of the disease, lengthen remission and improve quality of life and psychosocial functioning of patients.

The psychology of patients with IBD has been of great interest to many investigators [5-12]. A number of high quality studies have shown a link between the patient's psyche and the course of the disease [5,13,14]. However, when psychological treatment in the form of psychotherapy is given most randomized controlled trials in IBD patients have found it to be ineffective [15-19]. Thus, some investigators have proposed that treating psychological co-morbidities with antidepressants might be of more utility to control disease activity and lengthen remission, particularly in Crohn's disease patients [20-23]. This may be particularly likely because treatment with antidepressants has been found effective in irritable bowel syndrome (IBS) [24-27], a partly postinfectious disease that commonly co-exists with IBD [28,29]. Thus far, a systematic analysis of available studies assessing the efficacy of antidepressant therapy for the control of somatic symptoms in IBD patients has not been undertaken. Therefore, the purpose of this study is to examine whether the existing literature supports a role for antidepressants in managing inflammatory bowel disease.

## Methods

### Study selection

#### Inclusion criteria

All forms of research publications, describing any study design published between 1990 and 2005, which examined the use of antidepressants in inflammatory bowel disease, were included in this review. Participants had to have a diagnosis of inflammatory bowel disease (either Crohn's disease or ulcerative colitis or not specified inflammatory disorder). Studies published before 1990 were not included, as the researchers were mostly interested in the newest antidepressants, introduced to the market in the 1990s.

#### Primary outcome measure

Our primary outcome measure was the efficacy of antidepressants in maintaining or inducing remission of inflammatory bowel disease. Remission was defined as at least a 4-week period with disease activity at less than 150 points or a drop in a score of at least 70 points for Crohn's disease and a score < 2 points or a drop in a score of at least 3 points for ulcerative colitis, measured on the Crohn's Disease Activity Index and the Simple Clinical Colitis Activity Index, respectively OR at least a 4-week period with a complete lack of IBD symptoms [30,31]. Therefore, the effectiveness of treatment with antidepressants in IBD patients was defined as the remission of the disease activity beyond the usual length of remission (different for a particular patient).

### Data source

We electronically searched PubMed, CINAHL, Cochrane (Cochrane Depression, Anxiety and Neurosis Group and the Cochrane Inflammatory Bowel Disease and Functional Bowel Disorders group), PsycInfo, and Embase databases in July 2005 – September 2005 for any studies published between 1990 and 2005 in which antidepressants were used in inflammatory bowel disease patients. Follow-up searches were conducted in October and November 2005 to identify more recently published papers. We also conducted hand searches of the following journals: *Gastroenterology*, *Current Treatment Options in Gastroenterology*, *Psychosomatic Medicine*, and *General Hospital Psychiatry *in September – November 2005.

The search terms used were: Inflammatory Bowel Diseases OR Inflammatory Bowel Disease OR Colitis, Ulcerative OR Ulcerative Colitis OR Crohn Disease OR Crohn* Disease OR Granulomatous Colitis OR Granulomatous Enteritis OR regional Ileitis OR terminal Ileitis OR Ileocolitis OR ibd AND Antidepressive Agents OR antidepress* OR Thymoanaleptics OR Thymoleptics OR ssri OR mirtazapine OR bupropion OR paroxetine OR selective serotonin reuptake inhibitor* OR selective norepinephrine serotonin reuptake inhibitor*. There was no language restriction. We searched for additional studies in the reference lists of identified articles. We also searched unpublished theses stored at the University of Adelaide and in the whole of Australia through the National Library of Australia. We additionally contacted selected experts (18 South Australian gastroenterologists).

### Data extraction

The systematic review identified no randomized controlled trials, cohort prospective studies nor case-controlled studies. Nevertheless, we located an open-label study, case reports, reviews, a guideline, a discussion, and a letter. The quality of the selected studies was assessed using the Cochrane Reviewers' Handbook [32]. However, because all the identified studies were non-randomised we also used the appendix to this manual prepared by the Cochrane Non-Randomised Studies Methods Group [33]. The standard components of the quality assessment are: sample size, allocation concealment, clear description of treatment, representative source of subjects, use of diagnostic criteria or inclusion criteria, outcome measures described and the use of validated instruments. The Cochrane Non-Randomised Studies Methods Group has not specified the components of the quality assessment for non-randomised trials as yet. We therefore needed to create our own criteria. As our systematic review identified mainly case reports, reviews and one open-label study, we focused in assessing their quality on: length of a treatment, follow-up, clear description of a treatment, description of participants, and the use of validated instruments. We are aware of the various limitations of these studies. However, because no other published data exists in this field it is important to sum up the up-to-date literature as it may inform and facilitate future randomised trials in this area.

### Data synthesis

As statistical data were accessible only for one study, and even then were limited, we could not conduct a meta-analysis.

## Results

### Description of studies

We identified a total of 106 articles from the electronic databases, most of which were not directly related to the entered keywords. Only 12 publications met the inclusion criteria. During hand searches of journals, and during searches of reference lists of identified articles and unpublished theses we found no new articles that would match our inclusion criteria. The contact with selected experts also failed to identify new papers.

In order of design quality articles that met the inclusion criteria were: one non-randomised open-label trial, six case reports, three reviews, one guideline, one discussion paper, and one letter referring to previous studies. The total number of IBD patients described was only 20; eight participants were described in the open-label study and 12 in case reports. Antidepressants described included: paroxetine, bupropion, amitriptyline, phenelzine and mirtazapine. Paroxetine was used in three studies in 12 participants. Bupropion was used in two studies in six participants, and was recommended for use in IBD patients in two reviews, one discussion paper and one letter. Amitriptyline was used in one study in one participant. Phenelzine was used in one study in one participant. Mirtazapine was not recommended in one review (Table 1).

**Table 1:** Features of 12 studies describing the effect of antidepressants on the course of inflammatory bowel disease in order of the quality significance

Study name	Design	Participants	Disease type	Method	Results
Kast and Altschuler 2001 (USA)	Case report	2: Female, 44 y.o., 10 years with CD, CDAI: 202, mesalamine 500 mg (2 a day), once a year a relapse treated with steroids, depression treated with fluoxetine (40 mg) not effectively Male, 45 y.o., 20 years with CD, CDAI: 275, azathioprine (100 mg) and bowel resections, fluoxetine for pain not effective.	CD	Bupropion 150 mg (3 times daily) for depression (a female) and for pain and smoking cessation (a male)	Female:19-month remission, bupropion dependant, no other medication, CDAI = 0 Male: CDAI = 45, 3–4 diarhoeas daily because of ileal-cecal valve, 50 mg azathioprine, still on bupropion. **Positive effect of bupropion on IBD activity (CD).**
Walker et al. 1996 (USA)	Non-randomised open label study	8, recruited between March and October 1993 in tertiary care medical facility in Seattle, English-speaking, 18 y.o. or older, presented with IBD	Not specified	Tools: NIMH Diagnostic Interview Schedule (psychiatric interview), GI symptom interview and the Briere Child Maltreatment interview (history of childhood abuse and neglect), SF-36, Tridimensional Personality Questionnaire Patients diagnosed with major depression (n = 8) have their depression confirmed by the Hamilton Depression Inventory (HAM-D) and started treatment. Treatment: paroxetine (paxil) 20 mg, after 1 month two patients had the dosage increased to 40 mg. Length: 8 weeks and reinterviewed + SF-36 and HAM-D	Decrease in mean HAM-D (pre-treatment 29.0+-7.8; post-treatment 8.1+-6.1; t = 13.6, df = 7, p,0.0001) and significant reduction in functional disability on most scales of the SF-36. The SF-36 measures changes in several domains of patient function including physical limitations, occupational role, emotional role, social function, pain, mental health, vitality, and health perception (higher scores associated with increased quality of life) **Positive effect of paroxetine on IBD activity (not specified).**
Scott, Letrent, Hager and Burch 1999 (USA)	Case report	1, 42 y.o., black male, depressed with chronic abdominal pain, weight loss, insomnia, anhedonia, with flare of CD, taking 6-mercaptopurine, prednisone and total parenteral nutrition. Treated in the past for depression with sertraline ineffectively and with amitriptyline successfully.	CD	Amitriptyline gel 80 mg/day intramuscularly. Improvement in mood but not in pain. Then, transdermal gel Amitriptyline 150 mg applied to the chest at bedtime. Tool: Hamilton Depression Scale	Follow-up 6 weeks. Depression did not respond adequately to transdermal amitriptyline, however, patient stated that his mood improved. Patient's abdominal pain remained unchanged, however, did not experience any adverse events associated with transdermal medication. **No effect of amitriptyline on IBD activity (CD).**
Eirund 1998 (Germany)	Case report	1, male, 67 y.o., 17 years with UC, 4 relapses per year despite the treatment with sulfasalazine	UC	Treatment with paroxetine (20 mg) for panic disorder	Panic disorder cured. No relapse of UC for 10 months. **Positive effect of paroxetine on IBD activity (UC).**
Kast 1998 (USA)	Case report	1, female, 33 y.o., 18 years with CD, taking azathioprine (75 mg), prednisone (60 mg) and acetaminophen (3 tablets daily), 3 bowel resections, despite this in relapse	CD	Phenelzine treatment for anxiety-prominent major depressive episode (15 mg 3 times daily – 30 mg 3 times daily)	Depression cured. After 7 days of treatment bowel movements dropped from 10 to 3–4 per day, after the increase to 30 mg 1 bowel movement daily, depression responded, no cramps. Azathioprine and prednisone tapered off. Remission for 2 years until the treatment with phenelzine stopped. After 6 weeks since the stop relapse. **Positive effect of phenelzine on IBD activity (CD).**
Kane, Altschuler and Kast 2003 (USA)	Case report	4, (2 women, 2 unspecified)	CD	Treatment with bupropion (100 mg daily) for smoking cessation (2 women) and depression (2 unspecified)	CDAI<150 within 6 weeks (without a change in standard medication for IBD) **Positive effect of bupropion on IBD activity (CD).**
Torras Bernaldez et al. 2003 (Spain)	Case report	3 depressed patients, no IBD diagnosed before depression	-	Treatment with Paroxetine for depression	Patients present with chronic diarrhoea, 2 treated with corticosteroid + immunosuppressants, 2 diagnosed with CD, one with unspecified bowel disease. **Controversial paroxetine.**
Ginsburg et al. 2005 (USA)	Guideline	0	Not specified	NA	**All antidepressants recommended in irritable bowel syndrome recommended in IBD.**
Kast 2003 (USA)	Review	NA	CD	NA	**Bupropion recommended and mirtazapine not recommended**.
Kast and Altschuler 2004 (USA)	Letter	NA	CD	NA	**Bupropion recommended.**
Kast and Altschuler 2005 (USA)	Review	NA	CD	NA	**Bupropion recommended.**
Kast 2005	Discussion	NA	CD	NA	**Bupropion recommended.**

Legend:

CD – Crohn's disease

CDAI – Crohn's Disease Activity Index

HAM-D – Hamilton Depression Inventory

IBD – inflammatory bowel disease

NIMH – National Institute of Mental Health

SF-36 – Medical Outcome Short Form (36) Health Survey

UC – ulcerative colitis

y.o. – years old

### Positive impact of antidepressants on inflammatory bowel disease activity

Five research studies indicated a positive impact of the treatment with antidepressants on the inflammatory bowel disease activity. Four out of these five studies were case reports. One study out of the five was an open-label trial. There were 16 patients treated with antidepressants in these five studies. All of them benefited from this treatment. Among 16 patients treated with antidepressants, there were four females, two males, and in 10 the gender was not specified. Among these patients, seven suffered from Crohn's disease, one from ulcerative colitis, and 8 from unspecified inflammatory bowel disorder. All patients were adults. Examined antidepressants were: paroxetine (two studies), bupropion (two studies), and phenelzine (one study).

Two reviews, one discussion paper, and one letter also endorsed a positive impact of the treatment with antidepressants on the inflammatory bowel disease activity, despite the lack of provision of any new patient data. In all four of these papers bupropion was recommended for use in inflammatory bowel disease patients. In one paper, mirtazapine was not recommended for use in inflammatory bowel disease (See: Discussion for explanation).

### Negative impact or no impact of antidepressants on inflammatory bowel disease activity

One study, a case report, suggested a potential negative impact of an antidepressant on inflammatory bowel disease. Three patients were described, and the antidepressant used was paroxetine. Prior to paroxetine patients had not been diagnosed with inflammatory bowel disease. Following treatment with paroxetine for depression, patients developed chronic diarrhoea and were subsequently diagnosed with inflammatory bowel disease, Crohn's disease in two patients and non-specified inflammatory bowel disorder in one patient. There was no control group in the study however, and the significance of this observation seems unclear.

Another case report found no impact of antidepressants on inflammatory bowel disease. One adult, male participant was described, and the antidepressant used was transdermal amitriptyline gel. Amitriptyline did not help the patient with pain nor fully treat the depression, however, the patient noticed his mood improved.

### Quality of studies

We set five quality criteria for analysis: length of treatment with antidepressant, follow-up, clear description of treatment, description of participants, and validated instruments (See: Table 2).

**Table 2:** Quality assessments in order of the quality significance

	Kast et al. 2001	Walker et al. 1996	Scott et al. 1999	Eirund 1998	Kast 1998	Kane et al. 2003	Torras et al. 2003	Ginsburg et al. 2005	Kast 2003	Kast et al. 2004	Kast et al. 2005	Kast 2005
**Length of treatment**	2 years	8 weeks	6 weeks	10 months	2 years	No data	No	NA	NA	NA	NA	NA
**Follow-up**	Yes, regular follow-up for 2 years	Yes, 2 follow-up, all patients completed	Yes, detailed every day monitoring for 6 weeks.	1 follow-up after 10 months	Yes, regular follow-up for 2 years	1 follow-up after 6 weeks	Yes	NA	NA	NA	NA	NA
**Clear description of treatment**	Yes	Yes	Yes	Yes	Yes	Yes	No	Yes	Yes	Yes	Yes	Yes
**Description of participants**	Yes	Yes	Yes	Yes	Yes	No	No	NA	NA	NA	NA	NA
**Validated instruments**	Yes	Yes, but only for depression and quality of life	Yes (depression), No (CD)	No data	No	Yes	No	NA	NA	NA	NA	NA
**Limitations**	-	Disease type not specified, disease activity index not used.	Lack of CD activity index. Focus only on depression and pain, no information about frequency of stools.	No objective activity index used.	No objective activity index used.	Lack of explanation of patients characteristics, Lack of further follow-up, no length of treatment provided.	Lack of evidence that IBD did not exist before the onset of depression, no details about length of treatment, no description of treatment and participants, no information about instruments.	Guideline paper without research. IBD treated as IBS, which may not be appropriate as they are different conditions.	A review study without the research. Theoretical considerations only.	A letter referring to Kane et al. 2003	A review study without the research. Theoretical considerations only.	A discussion referring to Kast et al. 2001 and Kane et al. 2003
**Impact of treatment with antidepressants on the IBD activity**	Positive effect of bupropion on IBD activity (CD).	Positive effect of paroxetine on IBD activity (not specified).	No effect of amitriptyline on IBD activity (CD).	Positive effect of paroxetine on IBD activity (UC).	Positive effect of phenelzine on IBD activity (CD).	Positive effect of bupropion on IBD activity (CD).	Controversial paroxetine.	All antidepressants recommended in irritable bowel syndrome recommended in IBD.	Bupropion recommended and mirtazapine not recommended.	Bupropion recommended.	Bupropion recommended.	Bupropion recommended.

Legend:

CD – Crohn's disease

IBD – inflammatory bowel disease

IBS – irritable bowel syndrome

NA – not applicable

UC – ulcerative colitis

Five studies met all or four out of five of the quality criteria. One article met three out of five of the quality criteria. Six publications met only one out of five of the quality criteria. The study by Kast et al. (2001) met all the quality criteria chosen in this systematic review. The next best study was by Walker et al. (1996). However, the weakness of this study lay in the fact that the authors did not specify the IBD type and did not use a disease activity index. The study by Scott et al. (1999) also matched the majority of the quality criteria, however, the researchers did not use a validated instrument to measure the inflammatory bowel disease activity, and they focused only on pain and depression, as their intention was to treat these disorders, rather than to influence the IBD activity. Eirund et al.'s (1998) study addressed four out of five quality criteria, however, these researchers also failed to use an inflammatory bowel disease activity index. The study by Kast (1998) also met four criteria out of five, but again, the researchers did not use a validated instrument to measure the inflammatory bowel disease activity. Kane et al.'s (2003) study addressed three out of five criteria. The researchers did not provide the information about the length of treatment nor did they describe patients' characteristics and further follow-up. The non-experimental papers [34-38] and the study by Torras et al. (2003) did not match the quality criteria. Moreover, although their recommendations were of interest and may help in understanding the problem, they added no new data.

## Discussion

This systematic review is the first structured attempt to explore the hypothesis that the clinical course of IBD may be influenced by specifically treating psychological co-morbidities with antidepressants. Despite widespread acknowledgement that IBD is not a "6 week illness" [39] the psychological dimension of the illness has been largely ignored in standard treatment paradigms. Unfortunately, because of the paucity of published data – both quantitative and qualitative – this review is unable to provide a definitive answer on whether IBD can be improved by antidepressant therapy. However, it has highlighted a significant "evidence gap" in the literature, supporting our premise that psychological co-morbidities in patients with inflammatory bowel disease are often unrecognised and in general remain under-treated and under-researched.

While we acknowledge the poor methodological quality of the collected studies, they do however indicate that antidepressants appeared not only to help certain individual patients with IBD cope with their emotional problems, but also improved their quality of life. The published observations also hold out the intriguing possibility that antidepressant therapy may have specifically influenced the course of their inflammatory disease. This novel therapeutic possibility warrants further consideration.

To date, researchers with an interest in the impact of a patient's psyche on disease activity in IBD have conducted little formal research on the use of antidepressants in this condition, with most published data being uncontrolled and anecdotal. Only 12 relevant articles were identified, and five of these did not include new data. However, the three case reports [20-22], two reviews [35,38], and a discussion paper [37] did explore the potential for influencing disease activity in IBD with antidepressants from a novel perspective. Kast [21] presented a medical history of a depressed and anxious patient with Crohn's disease who was treated with phenelzine and subsequently achieved remission from IBD. In further reports, Kast and Altschuler [22] describe two additional patients who achieved long-lasting remission of Crohn's disease whilst using bupropion. These investigators hypothesize that this may have resulted from decreased tumor necrosis factor-alpha (TNFα), which is known to play a vital role in Crohn's disease. Both phenelzine and bupropion increase intracellular cAMP [40] which, in turn, decreases TNFα. As phenelzine may cause a hypertensive crisis, bupropion is suggested to be a safer therapeutic option. Interestingly, phenelzine and other monoamine oxidase inhibitors have also been noted to induce remission of rheumatoid arthritis, a disease in which – as in Crohn's disease – TNFα has a central role [41]. Kast [35], compared the use of bupropion and mirtazapine in patients with Crohn's disease. He speculated that both these antidepressants have the potential to affect inflammatory responses: bupropion by lowering TNFα and mirtazapine by increasing its level. Therefore, according to his hypothesis [35], there are theoretical reasons for recommending bupropion and cautioning against mirtazapine when treating depression in patients with Crohn's disease. Although Kast's explanations appear logical and are supported by other investigators [20], their practical effectiveness needs to be experimentally confirmed in appropriate clinical studies.

The largest study in this systematic review, which most closely matched the standard quality criteria, (See: Data extraction) was that by Walker, Gelfand et al. (1996). Whilst comparing IBD patients with and without current psychological disorders, the investigators noticed that depressed patients (n = 8) who were given paroxetine in an open label design showed significant improvement in relation to functional disability. The researchers had expected an improvement only in depression, however, patients' scores on SF-12 also improved in the area of "physical limitations, occupational role, emotional role, social function, pain, mental health, vitality, and health perception, with higher scores associated with increased quality of life" [23]. Although the importance of this result seems unquestionable, significant weaknesses of the study need to be acknowledged. These include the small sample size, open label design and the fact that the investigators did not differentiate between patients with Crohn's disease and ulcerative colitis. However, given the potential importance of this observation, larger studies addressing these methodological issues are clearly needed.

In discussing the possible effects of antidepressant therapy on IBD as revealed by this systematic review, we acknowledge that all the analysed studies were characterized by various limitations. Specifically these limitations included: lack of randomisation or control groups, potential lack of relevance to ulcerative colitis as most data pertain to Crohn's disease, non homogenous participants, failure to consistently apply standardised instruments, and lack of routine follow-up tests. Similarly, because of the uncontrolled nature of the observations, all studies are open to selection, performance, attrition and detection bias. Moreover, seven of 12 publications were from the same group, and four of these were discussions that did not contain any new data.

The fact that there is little good quality data in this area does not mean it is unworthy of further study. Our present state of knowledge regarding the interaction between psychological co-morbidities and IBD resembles that of our knowledge of the pathogenesis of irritable bowel syndrome twenty years ago. At that stage, IBS was thought to be predominantly a psychological condition, whereas we now have clear-cut, well accepted evidence of pathological abnormalities in the gut [42]. Currently IBD is generally thought to be purely inflammatory, but clinical observations in individual patients make it hard to dismiss the potential role of psychological factors [43]. Moreover, it is well accepted that IBS frequently co-exists in patients with IBD [28,29] and that both patient groups suffer similar impairment in physical and psychological domains [28], making rigid distinctions between psychological versus inflammatory mechanisms more difficult to uphold. As antidepressants are widely used in IBS [24-27,44] and because doctors already empirically treat depression, anxiety and pain in IBD using guidelines for IBS [34,43], creating informed discussion and hopefully prompting further research in this area is therefore likely to improve our understanding of IBD and patient care.

## Conclusion

Based on our findings, it is not possible to state that antidepressants have an impact on the course of inflammatory bowel disease. While they appeared to have a positive effect in 16 patients in five studies, because studies were neither randomised nor blinded, it can still be argued that the outcome may be due to chance. This systematic review therefore provides insufficient evidence to conclude that antidepressants are efficacious for the treatment of psychological co-morbidities or somatic complaints, which do not respond to conventional therapy in IBD patients. However, the data certainly do not rule out efficacy, and warrant further properly designed randomised controlled trials.

## Abbreviations

Inflammatory bowel disease (IBD); Crohn's disease (CD); ulcerative colitis (UC); irritable bowel syndrome (IBS)

## Competing interests

This study was funded by the Discipline of General Practice and School of Psychology at the University of Adelaide, and the Department of Gastroenterology, Hepatology and General Medicine at the Royal Adelaide Hospital from International Postgraduate Research Scholarship. No industry sponsorship was involved. However, Professor Deborah Turnbull would like to acknowledge that she has received research support from AstraZeneca Pty Ltd, Aventis Pharma Pty Ltd, Bayer Australia Ltd, and Pfizer Pty Ltd. Dr Jane M. Andrews would like to acknowledge that she has been a consultant for Schering Plough and Pharmatel Fresenius Kabi. Professor Gerald Holtmann would like to acknowledge that he has been a consultant for Abbott, Altana, AstraZenecka, Takeda, Janssen-Cilag, Steigerwald, Knoll, and Novartis. He has also received research support from Altana, Ardey Pharma, Deutsche Forschungsgemeinschaft, Zeria, and Novartis.

## Authors' contributions

AMW contributed to the conception of this manuscript, designed it, collected data, performed analysis and interpreted data, and wrote the first and final drafts.

DT contributed to the conception of this manuscript, revised it critically and gave the final approval of the version to be published.

NM revised the manuscript critically and contributed to the final draft.

IW revised the manuscript critically and contributed to the final draft.

JA revised the manuscript critically and contributed to the final draft.

GH revised the manuscript critically and contributed to the final draft.

All authors read and approved the final manuscript.
